# Quality Assessment of an Integrated Care Pathway Using Telemonitoring in Patients with Chronic Heart Failure and Chronic Obstructive Pulmonary Disease: Protocol for a Quasi-Experimental Study

**DOI:** 10.2196/20571

**Published:** 2020-11-19

**Authors:** Cyrille Herkert, Jos Johannes Kraal, Rudolph Ferdinand Spee, Anouk Serier, Lidwien Graat-Verboom, Hareld Marijn Clemens Kemps

**Affiliations:** 1 Flow, Center for Prevention, Telemedicine and Rehabilitation in Chronic Disease, Máxima Medical Center Eindhoven Netherlands; 2 Faculty of Industrial Design Engineering, Delft University of Technology Delft Netherlands; 3 Department of Cardiology, Máxima Medical Center Eindhoven Netherlands; 4 Department of Pulmonology, Máxima Medical Center Eindhoven Netherlands; 5 Department of Industrial Design, Eindhoven University of Technology Eindhoven Netherlands

**Keywords:** chronic heart failure, chronic obstructive pulmonary disease, integrated care pathway, telemonitoring

## Abstract

**Background:**

Chronic heart failure (CHF) and chronic obstructive pulmonary disease (COPD) often coexist and are associated with a high morbidity and reduced quality of life (QoL). Although these diseases share similarities in symptoms and clinical course, and exacerbations of both diseases often overlap, care pathways for both conditions are usually not integrated. This results in frequent outpatient consultations and suboptimal treatment during exacerbations, leading to frequent hospital admissions. Therefore, we propose an integrated care pathway for both diseases, using telemonitoring to detect deterioration at an early stage and a single case manager for both diseases.

**Objective:**

This study aims to investigate whether an integrated care pathway using telemonitoring in patients with combined CHF and COPD results in a higher general health-related QoL (HRQoL) as compared with the traditional care pathways. Secondary end points include disease-specific HRQoL, level of self-management, patient satisfaction, compliance to the program, and cost-effectiveness.

**Methods:**

This is a monocenter, prospective study using a quasi-experimental interrupted time series design. Thirty patients with combined CHF and COPD are included. The study period of 2.5 years per patient is divided into a preintervention phase (6 months) and a postintervention phase (2 years) in which end points are assessed. The intervention consists of an on-demand treatment strategy based on monitoring symptoms related to CHF/COPD and vital parameters (weight, blood pressure, heart rate, oxygen saturation, temperature), which are uploaded on a digital platform. The monitoring frequency and the limit values of the measurements to detect abnormalities are determined individually. Monitoring is performed by a case manager, who has the opportunity for a daily multidisciplinary meeting with both the cardiologist and the pulmonologist. Routine appointments at the outpatient clinic are cancelled and replaced by telemonitoring-guided treatment.

**Results:**

Following ethical approval of the study protocol, the first patient was included in May 2018. Inclusion is expected to be complete in May 2021.

**Conclusions:**

This study is the first to evaluate the effects of a novel integrated care pathway using telemonitoring for patients with combined CHF and COPD. Unique to this study is the concept of remote on-demand disease management by a single case manager for both diseases, combined with multidisciplinary meetings. Moreover, modern telemonitoring technology is used instead of, rather than as an addition to, regular care.

**Trial Registration:**

Netherlands Trial Register NL6741; https://www.trialregister.nl/trial/6741

**International Registered Report Identifier (IRRID):**

DERR1-10.2196/20571

## Introduction

### Background

Chronic heart failure (CHF) and chronic obstructive pulmonary disease (COPD) often coexist because they share similar risk factors, of which smoking and low-grade systemic inflammation are the most significant [1]. The prevalence of CHF in patients with COPD is approximately 20%, while the reported prevalence of COPD in patients with CHF ranges from 10% up to 50%, depending on the population studied and diagnostic criteria used to define COPD and CHF [1,2]. Both diseases share a chronic, progressive character and are associated with a poor quality of life (QoL) [3,4]. Health-related QoL (HRQoL) is negatively affected by the presence of comorbidities as well as frequent hospital admissions [3,5,6]. In fact, data from the Alliance for Home Health Quality and Innovation show a 60-day hospital readmission rate of 33% for elderly patients with combined CHF and COPD [7]. Obviously, this not only affects patient-related outcomes but it also causes a major economic burden. In addition to the overlap in pathophysiology, symptoms, and clinical course, CHF and COPD both require complex medication regimens associated with interactions between CHF and COPD medications. Concerning nonpharmacological treatment, both diseases require strict adherence to therapy and lifestyle recommendations. However, despite these similarities, in current daily practice, patients with CHF and COPD are treated in separate care pathways according to the CHF and COPD guidelines. Although these guidelines acknowledge the fact that diagnostic and therapeutic strategies should be integrated, no specific recommendations on integrated management are provided [8,9]. Recent studies show that there is a clear need for a more holistic approach for patients with chronic conditions [10,11]. In a survey among health care professionals, the following shortcomings in current COPD care pathways were identified: a lack of communication among health care providers, poor patient engagement, and a lack of a unified system targeting COPD and CHF [12]. Qualitative interviews in patients with CHF revealed that they experience a lack of cooperation among health care professionals in multiple health care sectors and insufficient patient education and counselling [13]. A qualitative study in patients with COPD showed that they struggle with the same problems as patients with CHF and that they opt for the introduction of a care coordinator and request self-monitoring and telehealth solutions [14].

Telehealth might provide a solution to these shortcomings. However, in 2017, only 40% of Dutch hospitals offered eHealth solutions to patients with CHF [15]. For patients with COPD, this is less well implemented. Worldwide, these percentages are even lower. The third global survey on eHealth, published in 2016 by the World Health Organization [16], showed that only 22% of the responding countries used an established remote patient monitoring solution. Telemonitoring studies on CHF as well as COPD show mixed results due to differences in telemonitoring interventions, study populations, and study end points [17,18]. Factors that might improve the effects of telemonitoring include personalization of telemonitoring regimes according to the patients’ needs, monitoring of comorbidities, and utilization of integrated care pathways rather than the application of telemonitoring as an add-on to regular care.

Given the overlap between CHF and COPD, the high costs of separate care pathways, and the call for a more holistic approach by patients and health care professionals, we propose a combined telemonitoring approach in an integrated care pathway. Unique to this approach is combined, on-demand disease management using modern technology (eg, face-to-face contact via videoconferencing) and delivering personalized care by one case manager for both diseases. Moreover, telemonitoring will be used instead of, rather than as an add-on to, regular care. In this way, telemonitoring is tailored to the patient’s demands, while covering more than one chronic disease.

### Objectives

The primary goal of this study is to investigate whether an integrated pathway using telemonitoring in patients with combined CHF and COPD results in a higher general HRQoL compared with traditional care pathways. Secondary end points include (1) disease-specific HRQoL, (2) level of self-management, (3) patient satisfaction, (4) compliance to the program, and (5) cost-effectiveness.

## Methods

### Study Design

This study was designed as a monocenter, prospective trial using a quasi-experimental interrupted time series design with a study period of 2.5 years. The study period starts with 6 months of regular care, after which the novel care pathway (see “Intervention”) will be introduced. End points are assessed monthly during these 2.5 years, resulting in 6 data points before introduction of the intervention and 24 data points after the start of the intervention. An interrupted time series design is the strongest quasi-experimental design to evaluate the longitudinal effects of interventions [19] and is more often used to evaluate the effects of interventions in the health care setting [20,21]. To achieve a balance between an acceptable wait time for the patient to start the intervention and to obtain sufficient preintervention data, a preintervention period of 6 months (6 data points) was chosen pragmatically.

**Figure 1:**
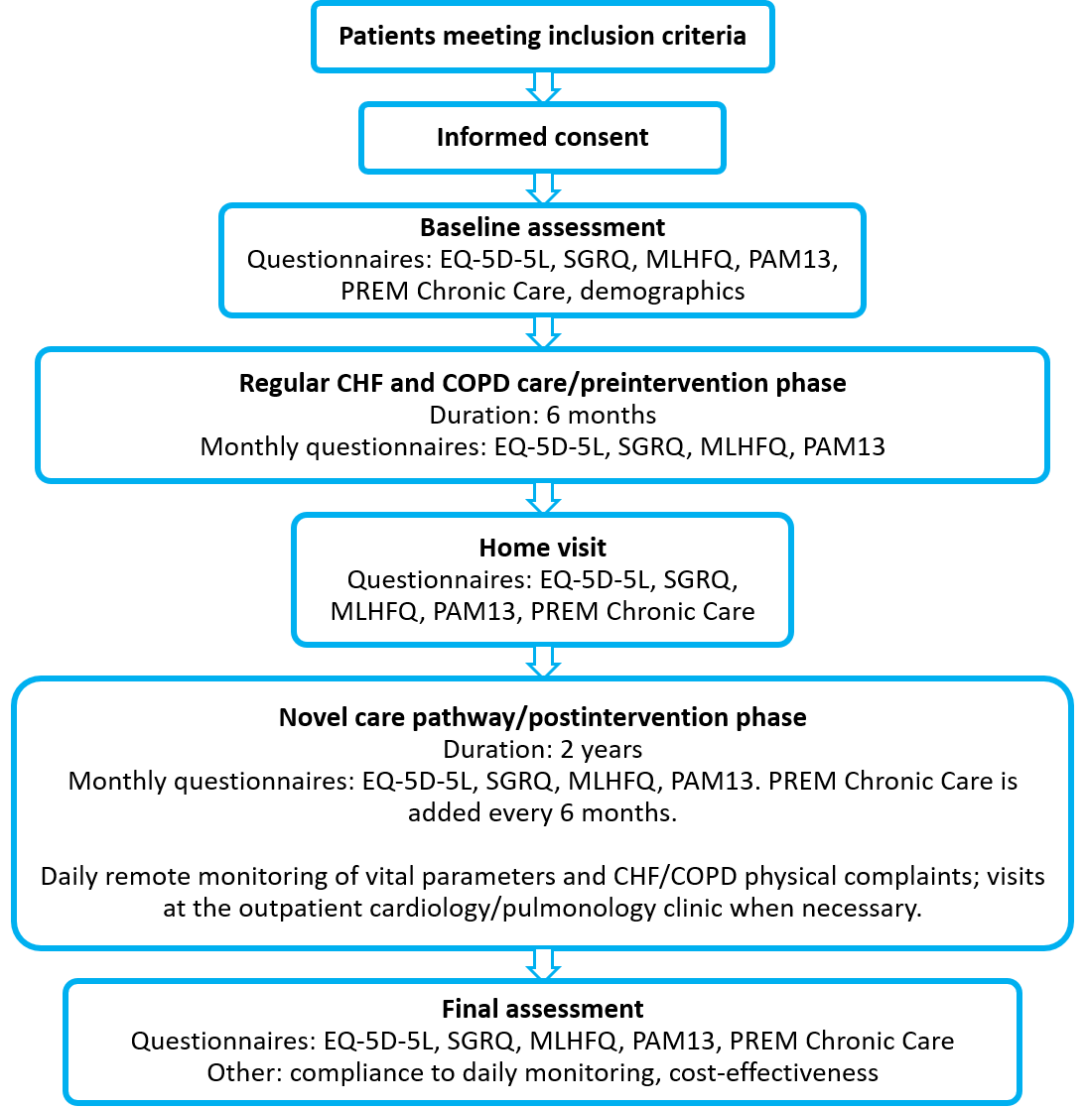
Flowchart of the study design. CHF: chronic heart failure; COPD: chronic obstructive pulmonary disease; MLHFQ: Minnesota Living with Heart Failure Questionnaire; PAM13: 13-item Patient Activation Measure; PREM: Patient-Reported Experience Measure; SGRQ: St George’s Respiratory Questionnaire.

An overview of the participants’ timeline and measurements is provided in Figure 1. The study will be performed in cooperation with the departments of cardiology and pulmonology at Máxima Medical Center in Eindhoven/Veldhoven, Netherlands.

### Eligibility Criteria

Eligible patients will be recruited by their cardiologist, pulmonologist, or CHF/COPD nurse, either at the outpatient clinic or at the cardiology/pulmonology ward. Patients (aged 16 years or older) who meet the diagnostic criteria for both CHF and COPD, regardless of etiology and severity (defined by the New York Heart Association [NYHA] class and the Global Initiative for Chronic Obstructive Lung Disease [GOLD] group, respectively), are considered for participation. CHF is defined as having symptoms that may be accompanied by signs that are caused by a structural and/or functional cardiac abnormality [8]. Patients will be included regardless of left ventricular ejection fraction (ie, diagnosed with heart failure with reduced ejection fraction [HFrEF], heart failure with preserved ejection fraction [HFpEF], or heart failure with mid-range ejection fraction [HFmrEF]).

COPD is defined as having symptoms and signs with matching spirometric findings [9]. Eligible patients should be treated for both CHF and COPD at Máxima Medical Center in Eindhoven/Veldhoven, Netherlands. Moreover, they must have been admitted to the hospital at least once during the previous year because of an exacerbation of CHF and/or COPD. Patients are required to have a personal computer, laptop, or tablet with an internet connection. Additionally, sufficient digital skills are needed. For patients without sufficient digital skills, informal caregivers could be involved to assist the patients. A complete list of inclusion and exclusion criteria can be found in Textbox 1. All subjects are requested to provide written informed consent before study entry (see Multimedia Appendix 1 for a sample informed consent form).

**Inclusion and exclusion criteria.**

Inclusion criteria

- Diagnosed with both chronic heart failure (heart failure with reduced ejection fraction, heart failure with mid-range ejection fraction, or heart failure with preserved ejection fraction) and chronic obstructive pulmonary disease, regardless of etiology.
- At least one hospital admission due to an exacerbation of chronic heart failure and/or chronic obstructive pulmonary disease during the last year.
- Aged 16 years or older.
- Able to speak and read the Dutch language.
- Life expectancy of more than 2.5 years.
- Sufficient digital skills (or assistance from an informal caregiver).

Exclusion criteria

- Not having an internet connection.
- Psychological disorders preventing the patient from study participation.

### Developmental Phase

The design of the intervention is based on the outcomes of a health care innovation project related to CHF and COPD care at Máxima Medical Center in Veldhoven, Netherlands. The aim of the project was to evaluate the current CHF and COPD care pathways and ideate new concepts to improve health care for these chronic patients. The results of this project were used to construct the current study.

The first part of the developmental phase consisted of two focus group meetings. Each group consisted of four patients who were diagnosed with combined CHF/COPD and had at least one hospital admission due to an exacerbation of CHF and/or COPD in the past year. Each patient was accompanied by a relative or spouse. The meetings were led by a specialized nurse. The interviews were aimed at revealing the most important themes and areas of concern, with respect to both the current care pathway and a future telemonitoring-guided care pathway. The main conclusions are summarized in Textbox 2.

The second part of this phase consisted of the assessment of the level of self-management and health care consumption of patients with combined CHF and COPD. To assess the level of self-management, the 13-item Patient Activation Measure (PAM13) was sent to 37 patients, of which 23 patients responded. The PAM13 reflects 4 levels of patient activation, where level 1 reflects the lowest level of self-management and level 4 reflects the highest level. The mean level of patient activation among these 23 patients, based on the PAM13 results, was 2, indicating that these patients lacked the knowledge and confidence to manage their diseases. An overview of the average annual health care consumption by these patients can be found in Table 1. Both the patients’ opinions and data on level of self-management and health care consumption were used to design the study intervention.

**Main conclusions of focus group meetings (n=8).**

Current care pathway

- Patients experience a lack of communication among different health care providers.
- More information about the use of medication, changes in medication, and knowledge about drug side effects and interactions is needed.
- Patients would like to learn how to better manage their chronic heart failure and chronic obstructive pulmonary disease.

Future telemonitoring-guided care pathway

- Patients would appreciate a quick intervention for an upcoming exacerbation.
- It would be beneficial to have one contact person for both diseases.
- Daily monitoring would provide a feeling of safety and prevent patients from waiting too long before consulting a health care professional for their chronic heart failure/chronic obstructive pulmonary disease–related physical complaints.
- Daily monitoring would provide feelings of safety; however, when clinically stable, the monitoring frequency should be reduced to gain trust in their own body.

**Table 1:** Average annual health care consumption by patients with combined chronic heart failure (CHF) and chronic obstructive pulmonary disease (COPD) (n=23).

	Number/year
Hospital admissions	2.3
Days admitted to the hospital	14.6
Visits to an emergency department	0.3
Visits to a cardiologist/pulmonologist	5.6
Telephonic appointments with a cardiologist or pulmonologist	1.7
Visits to a CHF/COPD nurse	2.1
Telephonic appointments with a CHF/COPD nurse	1.6
Cardiac examinations (electrocardiogram, ultrasound, Holter monitoring)	4.4
Spirometry	2.0

### Usual Care

During the first 6 months of the study period, patients receive regular care by their cardiologist, pulmonologist, and CHF or COPD nurse (ie, by appointments at the outpatient clinic).

### Intervention

The intervention starts with a home visit by the patient’s case manager (a specialized CHF/COPD nurse). The major goals of this home visit are (1) to familiarize themself with the patient, (2) to get an impression of the home environment of the patient, (3) to take a cardiac and pulmonary anamnesis and to evaluate the medication use, (4) to provide the patient with instructions for the use of the sensors and digital platform, and (5) to involve informal caregivers in the telemonitoring process (if needed).

During the home visit, the following sensors are provided to the patient: a blood pressure monitor (iHealth Track, iHealth Labs Inc), a pulse oximeter (iHealth Air, iHealth Labs Inc), a weighing scale (iHealth Lite, iHealth Labs Inc), and a thermometer (Thermoval Basic, Hartmann BV). In addition, participants are instructed on the use of a personalized and secured digital platform (Mibida BV). Safety and privacy are warranted by using encryption and signature layers. To improve usability, the platform was developed and tested before the start of the study. This was done together with a patient with CHF and COPD. For patients, the platform is equipped with the following functionalities:

- daily entry of vital parameters—although there is a possibility for automatic transfer of iHealth sensor data to the digital platform (via Bluetooth), study participants enter their data manually. This is to assure data safety, as storing medical data on the iHealth app and corresponding cloud is undesirable. Moreover, dealing actively with measured parameters, by reading numbers on the sensors and then manually entering them on the digital platform, might enhance insight into their own vital parameters and promote self-management.
- daily entry of physical complaints related to CHF and COPD—the questionnaire consists of 5 short questions. If a patient answers “yes” (eg, to the question about whether they suffer from coughing), additional questions appear to get a more complete overview of the patient’s clinical status and to try to differentiate between CHF- and COPD-related physical complaints.
- a graphical overview of measured vital parameters over periods of time.
- a modality for videoconferencing with the case manager.
- a modality to send chat messages to the case manager.
- a tab with reports made by the case manager (eg, to refer to changes in medication being made).
- a tab with upcoming appointments (ie, video calls or appointments at the outpatient clinic, if needed).

During the first month of the intervention, all participants are instructed to complete the short questionnaire on physical complaints related to CHF and COPD and to perform sensor measurements (blood pressure, heart rate, weight, oxygen saturation, and body temperature) on a daily basis. For each measured vital parameter, personalized reference values are set, categorizing values into normal, slightly abnormal, or abnormal. These reference values are set in a multidisciplinary setting with the case manager, cardiologist, and pulmonologist. Case managers perform daily reviews (except during the weekends) based on a list of all participants. This list gives a practical overview of the clinical status of each patient. All measurements that raise an alarm, or questionnaires that indicate physical complaints, are indicated in the list of participants. The patient with the most alarming parameters appears at the top of the list. If a patient appears to be clinically unstable based on the parameters, a video call will take place to get a more complete overview of the patient’s status. Moreover, the patients are provided with a central phone number to get in touch with the case manager when experiencing acute physical complaints.

Every afternoon, a multidisciplinary meeting of the cardiologist, pulmonologist, and case manager is scheduled to discuss patients that need supervision and to define their treatment strategy. After the first month, the measurement frequency is personalized to the patient depending on his or her clinical stability and preferences. Once every three months, the case manager will have a more extensive video call with the patient to discuss, for example, medication use, lifestyle recommendations, self-management skills, etc.

Regular appointments at the outpatient clinic with the cardiologist, pulmonologist, and CHF/COPD nurse do not take place during the postintervention phase. Appointments will only be planned if deemed necessary, based on the telemonitoring parameters or on the patient’s medical history. Thus, telemonitoring will not be used as an addition to regular care but rather will replace regular care, fitting with the patient’s needs.

### Outcomes

The primary outcome measure is general HRQoL. The secondary outcome measures are disease-specific HRQoL, level of self-management, patient satisfaction, compliance to the telemonitoring program, and cost-effectiveness. General HRQoL, disease-specific HRQoL, and level of self-management are assessed monthly during the entire study period (both preintervention and postintervention). Patient satisfaction of chronic care is assessed at baseline and then every 6 months. All questionnaires used to assess the end points will be sent to patients via email, using a web-based database software program (Castor Electronic Data Capture, Ciwit BV). Compliance to the telemonitoring program and cost-effectiveness will be assessed at the end of the study period.

#### General HRQoL: EQ-5D-5L

The primary outcome measure is general HRQoL. To measure general HRQoL, the validated questionnaire EQ-5D-5L is used. The EQ-5D-5L consists of 5 questions in 5 domains and a visual analog scale to grade QoL. It is a valid and responsive measure of health status in both patients with respiratory disease and patients with cardiovascular disease [22,23]. This questionnaire will also be used in cost-effectiveness analysis by calculating quality-adjusted life-years (QALYs).

Due to the progressive nature of both CHF and COPD, a gradual decline in HRQoL is expected. We therefore hypothesize that the intervention will result in a reduced decline or improvement of HRQoL as compared with the preintervention phase. This study primarily focuses on HRQoL, as patient value in chronic care and research is often underrecognized.

#### Disease-Specific HRQoL: Minnesota Living with Heart Failure Questionnaire (MLHFQ) and St George’s Respiratory Questionnaire (SGRQ)

In addition to general HRQoL, disease-specific HRQoL will be assessed to evaluate the effect of both CHF and COPD on patients’ QoL. Heart failure–specific QoL is assessed by the MLHFQ. This questionnaire consists of 21 items, rated on 6-point Likert scales. The MLHFQ provides a total score for HRQoL, as well as subscores in the physical and emotional domains. The commonly used MLHFQ has shown good psychometric properties [24].

The SGRQ is a widely used and validated questionnaire among patients with COPD [25,26]. It consists of 50 items, some of which are scored on a Likert scale and others dichotomously. The SGRQ results in a total score and three subscale scores (symptoms, activity, and impact).

#### Level of Self-Management: PAM13

Self-management is assessed by the previously validated PAM13 [27,28]. PAM13 is a 13-item questionnaire scored using 4-point Likert scales. The PAM13 reflects 4 levels of patient activation. Level 1 reflects a passive role and a lack of confidence in maintaining or improving health status, while level 4 reflects the ability to adopt and maintain new behaviors.

#### Patient Satisfaction: Patient-Reported Experience Measure (PREM) Chronic Care

Patient experiences are assessed using the PREM Chronic Care questionnaire. This questionnaire was developed by InEen, Patiëntenfederatie Nederland, and Zorgverzekeraars Nederland and is designed for quality improvement of chronic integrated care. It is a validated 26-item questionnaire with different types of questions (ie, multiple choice, open-ended, 6-point Likert scale) about the patient’s experience with chronic care [29]. The PREM is sent to the patient at baseline and then every 6 months during the study period.

#### Compliance

Compliance to the telemonitoring program will be evaluated at the end of the study by reporting the percentage of completed monitoring sessions. A monitoring session comprises uploading the requested vital parameters and the corresponding questionnaire on physical complaints. An adherent patient is defined as a patient who completes at least 80% of the requested monitoring sessions. A nonadherent patient completes less than 20% of the requested number of monitoring sessions. A partially adherent patient completes 20% or more but less than 80% of the requested number of monitoring sessions. This classification is based on previous literature on adherence to a therapeutic regimen [30].

#### Cost-Effectiveness

For the cost-effectiveness analysis, the effects of the period before and after the intervention will be compared and related to their difference in costs. The effect is expressed in quality-adjusted life-years (QALYs) and will be calculated using index values derived from the monthly administered EQ-5D-5L [31]. The costs and QALYs of the preintervention period (6 months) will be compared with the monthly data from the first 6 months after the intervention has started. Only the first 6 months postintervention will be taken into account to enhance the quality of the comparison with the preintervention period. Although costs could be higher in the first 6 months postintervention because of training and set-up, QoL deteriorates over time in patients with COPD and CHF. Therefore, we decided to analyze only the first 6 months postintervention. In a sensitivity analysis, we will calculate the costs and QALYs for the whole postintervention period and calculate the monthly average for comparison with the preintervention period. The economic evaluation will be performed from a health care perspective. In-hospital costs will include telephonic and face-to-face contacts with the cardiologist, pulmonologist, heart failure nurse and pulmonary nurse, as well as ambulance transports, emergency department visits, hospital admissions, and associated in-hospital examinations (ie, electrocardiogram, cardiac ultrasound, spirometry, etc). These data are derived from the electronic patient files. Face-to-face and telephonic consultations with the general practitioner (GP) or nurse practitioners will be gathered by contacting the GP. The pharmacist will be asked to provide an overview of delivered medications for CHF and COPD. Prices will be gathered using the Dutch Manual for Costing in economic evaluations and market prices [32], the Dutch Pharmacotherapeutic Compass [33], and the financial division of the hospital. Costs related to the intervention include costs for the sensors, digital platform, home visit, telephonic and video calls, and costs associated with a multidisciplinary meeting.

### Sample Size

A power calculation is not possible because of the study design. Previous research tried to provide guidance on sample size calculation in an interrupted time series design, which appeared to be complex and needed various factors to be considered, such as the expected effect size, the location of the intervention in the time series, and the sample size per time point [34]. For this study, we chose a sample size of 30 participants. This was a pragmatic choice, based on the expected inclusion rate and the expected large effect size. The effect size is expected to be large because patients with CHF and COPD have a low QoL [35,36], and the study intervention is particularly designed to meet the patients’ needs and preferences (via interviews).

### Statistical Analysis

Descriptive statistics will be used to describe the population with regard to baseline characteristics and demographics. Continuous data will be presented as mean (SD) if normally distributed. Categorial data will be presented as n (%). For each outcome measure (eg, general HRQoL, disease-specific HRQoL, and level of self-management), each participant will create 6 data points before and 24 data points after the start of the intervention. Patterns in these data points will be analyzed using segmented regression analysis [19]. Regression estimates will be used to express the level and trend (ie, slope) of the outcome measures in both the preintervention phase and the postintervention phase in relation to the intervention. The presence of autocorrelation will be tested using the Durbin-Watson statistic [37]. If autocorrelation is present, we will adjust for it. Moreover, we will adjust for seasonality and time between last hospital admission and inclusion in the study. Missing data points will be imputed. Analysis will be performed using the statistical software package SPSS (version 26.0; IBM Corp).

## Results

### Ethics Approval

The study and its amendments were approved by the local ethics committee of Máxima Medical Center (see Multimedia Appendix 2 for the primary medical ethical approval form). The study will be conducted in accordance with the declaration of Helsinki and is registered at the Netherlands Trial Register (NTR) with registration number NL6741.

### Trial Status

Following ethical approval of the study protocol, the first patient was included in May 2018. Inclusion is expected to be complete in May 2021. With a study period of 2.5 years per patient, data collection is expected to be complete in November 2023.

## Discussion

The aim of this study is to improve the QoL of patients with combined CHF and COPD by introducing a novel, integrated care pathway using a remote, on-demand treatment strategy that was designed with input from patients. By providing feedback on the patient’s health status on a regular basis, improved levels of self-management are expected. This novel care pathway might lead to a higher efficacy in chronic care for patients with combined CHF and COPD and might therefore be accompanied by a reduction in health care costs.

Previous studies on telemonitoring in patients with CHF or COPD, which mainly focused on hard end points such as (re)hospitalization or mortality, showed conflicting results [38,39]. To improve the effectiveness of telemonitoring, we designed a novel intervention with several distinct features. First, telemonitoring will not be used as an add-on to regular care but rather will be incorporated in a well-defined care pathway. In this way, we aim to improve the cost-effectiveness of telemonitoring as compared with the interventions used in previous studies [40]. Second, the intervention is tailored to the disease stage. An unstable patient population at high risk of hospital readmission was selected using a telemonitoring intervention, which focuses on detecting clinical deterioration by daily monitoring of vital parameters and physical complaints. Clinically stable patients with CHF and COPD, without previous hospitalizations and with a low disease burden, might not benefit from a telemonitoring intervention focused on vital parameters; instead, these patients might benefit from a telemonitoring intervention focused on education and self-management, and replacing regular hospital visits with e-consultations. Finally, the intervention will target both CHF and COPD by assigning a case manager for both diseases. To our knowledge, this is the first study evaluating the effects of combined disease management using telemonitoring. Bernocchi et al [41] evaluated the effects of a novel care program for patients with combined CHF and COPD, but the program primarily focused on telerehabilitation. As mentioned previously, there is a need for a more holistic approach for patients with multiple chronic conditions. Therefore, future chronic care should focus not only on combined monitoring of two chronic conditions, but also on other prevalent chronic conditions, such as diabetes mellitus and chronic kidney disease.

### Conclusion

The remote patient monitoring CHF/COPD trial will provide important insights into the effects of a novel, combined care pathway for patients with CHF and COPD. Unique to this novel care pathway is that it is designed with the input of patients. Moreover, it offers disease monitoring using modern technology, delivering care on demand, having one case manager for both diseases, and providing an improved collaboration between the cardiologist and the pulmonologist.

## Appendix

Multimedia Appendix 1 Informed consent form.

Multimedia Appendix 2 Primary decision form of the medical ethical committee.

## Abbreviations

CHF: chronic heart failure
COPD: chronic obstructive pulmonary disease
GOLD: Global Initiative for Chronic Obstructive Lung Disease
GP: general practitioner
HFmrEF: heart failure with mid-range ejection fraction
HFrEF: heart failure with reduced ejection fraction
HFpEF: heart failure with preserved ejection fraction
HRQoL: health-related quality of life
MLHFQ: Minnesota Living with Heart Failure Questionnaire
NYHA: New York Heart Association
PAM: Patient Activation Measure
PREM: patient-reported experience measure
QALY: quality-adjusted life-year
QoL: quality of life
SGRQ: St George’s Respiratory Questionnaire
