# Supplementary material for: Quality Assessment of an Integrated Care Pathway Using Telemonitoring in Patients with Chronic Heart Failure and Chronic Obstructive Pulmonary Disease: Protocol for a Quasi-Experimental Study
Source: JMIR Res Protoc. 2020 Nov 19;9(11):e20571. doi: 10.2196/20571 (PMC7714643; doi:10.2196/20571)
Supplement: Multimedia Appendix 1 [file resprot_v9i11e20571_app1.docx]

## Multimedia appendix 1. Informed Consent Form

*Telemonitoring in patients with heart failure and COPD.*

- I have read the information letter, version 4.0 October 2019. I was also able to ask questions. My questions have been answered sufficiently. I have had enough time to decide whether or not to participate.
- I understand that participation is voluntary. I also know that I may decide at any time to not participate or to stop participating in the study. Without having to provide any reason.
- I give consent for my GP to be informed of my participation in this study.
- I know that for study purposes some individuals could have access to all my data. Those people are listed in this information letter. I consent to that access by these persons.
- I give consent to collect and use my data for answering the research question in this study
- I give consent to store my data at the research location for 15 years after completion of this study.
- I □ **give**

□ **do not give**
consent for the further storage of my personal data and retention for future research into the area of my diseases.

- I □ **give**

□ **do not give**consent to being contacted again after this study for a follow-up study.

- I want to participate in this study.

Name subject:

Signature: Date: __ / __ / __

-----------------------------------------------------------------------------------------------------------------

I certify that I have fully informed this subject about the study.

If information becomes known during the study that could influence the consent of the subject, I will

inform him/her of this on time.

Name investigator (or his/her representative):

Signature: Date: __ / __ / __
