# Supplementary material for: Quality Assessment of an Integrated Care Pathway Using Telemonitoring in Patients with Chronic Heart Failure and Chronic Obstructive Pulmonary Disease: Protocol for a Quasi-Experimental Study
Source: JMIR Res Protoc. 2020 Nov 19;9(11):e20571. doi: 10.2196/20571 (PMC7714643; doi:10.2196/20571)
Supplement: Multimedia Appendix 2 [file resprot_v9i11e20571_app2.pdf]

medisch ethische toetsingscommissie (METC)
secretariaat
telefoonnummer: 040888 9528
email: metc@mmc.nl

Mrs. drs. C. Herkert
Dept. Cardiology
Máxima MC
Location Veldhoven

Date:	22nd April 2020
Letter no.:	2020-084
Conc.:	Primary decistion (English version)
Study:	IN-TOuCH: Quality assessment of an integrated care pathway using telemonitoring in patients with chronic heart failure and COPD
METC-no.:	W18.002
CCMO-no.:	NL64413.015.17

We kindly ask you to use above mentioned METC-number in your future correspondence.

Dear mrs Herkert,

As requested, I hereby send you the English version of the decision of the research file of the above mentioned study as decided by the METC (Medical Ethical Reviewing Board) Máxima Medical Centre.

Kind regards,

Yolanda I.C. de Haan
Secretary METC

Decision
Primairy review

NL no.	NL64413.015.17	METC no.	W18.002
Title study	IN-TOuCH: Quality assessment of an integrated care pathway using telemonitoring in patients with chronic heart failure and COPD		

Contact details: *Mrs. C. Herkert, Máxima Medical Centre Veldhoven*
Sponsor: *Máxima Medical Centre Veldhoven.*

Decision
The medical ethical reviewing committee *Máxima Medisch Centrum* has reviewed the above-mentioned research file on the grounds of section 2, paragraph 2, sub a of the Medical Research Involving Human Subjects Act (WMO).

The committee hereby approves the research file, which as stated in the study protocol will be carried out in the following centre:
- *Máxima Medical Centre Veldhoven* – mr. dr. H.M.C. Kemps

Documents
The decision is based on the documents mentioned in appendix 1.

Background
On 21 December 2017 the research file was submitted to the METC Máxima Medical Centre for review. The research file was discussed during the plenary meeting that took place on 9th January 2018; see appendix 2 for the composition of the committee at this meeting.
An answer to our question letter and/or request for additional information from the METC of 10-01-2018was received on 30-01-2018.

Grounds
The METC Máxima Medisch Centrum is of the opinion that the condition(s), as stated in section 3 of the WMO, have been met. The main points of discussion were the objectives, the information letter for the subjects and the CE-marking of the used devices.

The committee has seen the enclosed investigation statements and is of the opinion that the conditions of the WMO, as stated in section 3, part f, have been met.

The committee is of the opinion that the conditions as stated in section 6, fifth till ninth paragraphs, of the WMO have been met. The research subjects and/or those persons authorized to give consent on their behalf for participating in the study, will be supplied with adequate, full and clear written information on the study.

Insurance
The committee has determined that the conditions regarding insurance have been met. The obligation to take out research subject insurance as stated in section 7, first paragraph of the WMO, has been correctly fulfilled. This is further specified in the document Medical Research (Human Subjects) Compulsory Insurance Decree 2015 (Decree, of 24th November 2014). The study falls under the research subject insurance of Máxima Medical Centre (MediRisk).

The committee has determined that the obligation to take out liability insurance as stated in section 7, ninth paragraph of the WMO, has been met.

Finally, the METC Máxima Medisch Centrum refers you to the conditions and obligations as stated in appendix 3.

Sincerely,
on behalf of the METC Máxima Medical Centre
Prof. dr. ir. H.L. Vader, Chair

Mrs. Yolanda I.C. de Haan, secretary medical ethical reviewing committee
Veldhoven, 1st February 2018 (original date Primairy review)
Veldhoven, 22nd February 2020 (translation Primairy review)

Appeals procedure
An appeal to this decision may be submitted within six weeks of the publication of the decision to the Central Committee on Research Involving Human Subjects (CCMO), on the grounds of section 23 of the WMO. The appeal should be addressed to: CCMO, PO Box 16302, 2500 BH The Hague, The Netherlands.

Appendix 1

Documents research file

A1. Cover letter [*21-12-2017*];
A1. Correspondence: query letter METC [*10-01-2018*] with letter registration 2018-003, answer letter submitter [*30-01-2018*];
B1. ABR-form [*version 2, 29-01-2018*];
B2. Local addendum [*19-12-2017*];
C1. Research protocol [*version 2.0, January 2018*];
D6. Instruction manual iHealth Wirdeless Blood Pressure Monitor;
D6. Instruction manual iHealth Wireless Pulse Oximeter;
D6. Instruction manual iHealth Wireless Body Analysis Scale;
D6. Instruction manual Thermoval standard;
D6. Instruction manual Fitbit Charge 2 (version 1.1);
D6. Sananet Care B.V. [ISO/IEC 27001:2013 certificat];
D6. Sananet Care B.V. [NEN 7510:2011 certificat];
D6. MiBida Security Paper;
D6. Medical App Checker [digital platform];
D6. Medical App Checker [iHealth App];
E1/2. Written information for the research subjects and/or for their lawful representatives, including consent form [*version 2.0, January 2018*];
F1. Questionnaires [*EQ-5D-5L, PAM-13 NL, SGRQ, COACH, PREM chronical care description*];
G1. WMO-research subject insurance [Máxima Medical Centre]:[policy number *AB-1000103 insurance company MediRisk*];
G2. Evidence of liability coverage of Stichting Máxima Medical Centre [*AB-1000103 of insurance company MediRisk*];
H1. CV of the independent expert [mr. *dr. A.G. Lieverse*];
I3. CV of the principal investigators [*dhr. dr. H.M.C. Kemps, mrs. C. Herkert*];
K6. Other documents [letter general practitioner].

Appendix 2

Members METC Máxima Medisch Centrum

The following members were present during the committee meeting on 9th January 2018:

- prof. dr. ir. H.L. Vader, clinical chemicus n.p., chair
- dr. H.P.J. Kemps, cardiologist, vice chair
- dr. H.J. Niemarkt, pediatrician-neonatologist
- dr. H.P.J. Willems, internist-immunologist-allergist
- dr. J.P. Dieleman, methodologist
- A.M.E. Hulshof, research subjects member
- dr. A.J.G. Maaskant, surgeon
- mr. R. Talma, lawyer
- dr. A.J.M. Oerlemans, ethicist
- dr. J.O.E.H. van Laar, gynaecologist
- dr. ir. C. van Pul, clinical physicist
- drs. B. Vugs, psychologist (written review)

Appendix 3

Conditions and obligations*

- **Validity decision**
  The positive decision loses its validity if the first participant/patient has not been included within one year of the decision being made
- **Amendments**
  Amendments are to be submitted to the METC Máxima Medisch Centrum
- **Start date study**
  The METC Máxima Medisch Centrum is to be informed of the actual start date of the study. This is the date on which the inclusion of the first patient has taken place.
- **Progress report**
  One year after the date of the decision being made, and every consecutive year thereafter, the METC is to be informed of the progress of the study by way of the form 'Voortgangsrapportage' (progress report)
- **Validity insurance**
  In the case of the insurance policy losing its validity during the course of the study, the METC Máxima MC is to receive a copy of the new valid insurance policy as soon as is possible.
- **Report in case of section 10**
  In the event of the trial proving to be significantly less beneficial to the subject than the research protocol has suggested, the METC Máxima Medisch Centrum is to be informed immediately along with a request for a further review.
- **Reporting SAEs**
  SAEs are to be reported to the METC Máxima Medisch Centrum
- **Reporting (premature) termination**
  (Premature) termination of the study is to be reported to the METC Máxima Medisch Centrum.
- **Final report**
  The METC Máxima Medisch Centrum is to be informed of the results of the study by way of a final report.

* *The legal timeframes and other information regarding submission of the various documents to the METC Máxima Medisch Centrum can be found on the website of the CCMO on the page 'Investigators'.*
